# The Influence of Ag Addition and Different SiO_2_ Precursors on the Structure of Silica Thin Films Synthesized by the Sol–Gel Method

**DOI:** 10.3390/molecules29194592

**Published:** 2024-09-27

**Authors:** Anna Adamczyk, Tomasz Brylewski, Patryk Szymczak

**Affiliations:** Faculty of Materials Science and Ceramics, AGH University of Kraków, Al. Mickiewicza 30, 30-059 Kraków, Poland; brylew@agh.edu.pl (T.B.); pszymcza@agh.edu.pl (P.S.)

**Keywords:** sol–gel method, silica coatings, TEOS, DDS, IR, SEM, AFM, GID, hydrophobicity, hydrophilicity, Ag addition

## Abstract

In this work, the structure of silica thin films synthesized with three different SiO_2_ precursors and obtained by the sol–gel method and dip coating technique was studied. Additionally, the influence of Ag addition on the obtained silica sols and then gel structure was investigated. Silica coatings show antireflective properties and high thermal resistance, as well as hydrophobic or hydrophilic properties. Three different silica precursors, TEOS (tetraethylorthosilicate), DDS (dimethyldietoxysilane) and Aerosil^TM^, were selected for the synthesis. DDS added to silica sol act as a pore size modifier, while Ag atoms are known for their antibacterial activity. Coatings were deposited on two different substrates: steel and titanium, dried and annealed at 500 °C in air (steel substrate) and in argon (titanium substrate). For all synthesized films, IR (infrared) spectroscopic studies were performed together with GID and XRD (Grazing Incidence Diffraction, X-ray Diffraction) measurements. The topography and morphology of the surface were traced by SEM and AFM microscopic methods, providing information on the samples’ roughness, particle sizes and thickness of the particular layers. The wetting angle values were also measured. GID and XRD measurements pointed to the distinct contribution of an amorphous phase in the samples, allowing us to recognize the crystalline phases and calculate the silver crystallite sizes. The FTIR spectra gave information on the first coordination sphere of the studied samples.

## 1. Introduction

Silica thin films deposited on various substrates by the sol–gel method have been investigated since the 1930s because they are easy to obtain and have advantageous properties [1]. They can play the role of high-thermal-resistance layers [2], antireflective coatings [3] or stabilize metal nanoparticles on glass surfaces [4]. Such coatings usually show good adhesion to the substrate, good tightness and low chemical reactivity. All these features ensure their high barrier properties. Silica thin films can be deposited on different types of substrates, e.g., metal alloys, metals, and glass, with no shape or dimensional limits.

The selection of the sol–gel method for synthesis offers a lot of benefits, such as the easy manipulation of chemical composition and the 100% usage of components that are of high purity. The reactivity of raw materials can be controlled by acid or basic catalysis and allows us to obtain samples of high homogeneity. The applied components allow the temperature of synthesis to be decreased and make it possible to avoid the necessity of using expensive equipment. Thus, this method is beneficial from an economic point of view. Unfortunately, it is also difficult to avoid the disadvantages of the sol–gel method, such as problems with repeatability and obtaining materials showing exactly the same properties, as well as the high sensitivity of this method to the synthesis conditions.

The sol–gel method allows materials based on silica matrix and featuring new properties tailored to their applications to be developed by including additional components. Silica-based materials containing silver are known to be perfect antibacterial materials, in the form of bulk samples or as thin films deposited on different types of substrates [5,6,7]. Silver at the nanoscale shows high biocompatibility and excellent resistance to sterilization conditions. Moreover, silver nanoparticles effectively block the growth of bacteria and are slowly released from the structure of a material.

Silica precursors, including DDS (CH_3_)_2_(C_2_H_5_O)_2_Si, play an important role as silica framework builders. DDS belongs to the group of polysiloxanes with a characteristic ladder structure. The addition of DDS to sols containing other silica precursors allows materials with a dense silica network but enhanced thermal resistance and less cracking to be obtained [8].

The application of three totally different types of silica precursors in the synthesis of silica sols/gels in the present work allowed us to observe their influence on the structure and selected properties of coatings and bulk samples. In studies concerning the dependence of the structure of silica gels on the silica precursor type applied in the sol–gel method, usually, two or more different but similar types of SiO_2_ precursor are taken into account, e.g., [9,10]. In contrast, in the present investigations, another component, namely nanoscaled silver, was used as a parameter influencing the structure of the synthesized samples.

## 2. Results

### 2.1. X-ray Diffraction Studies

**Figure 1:**
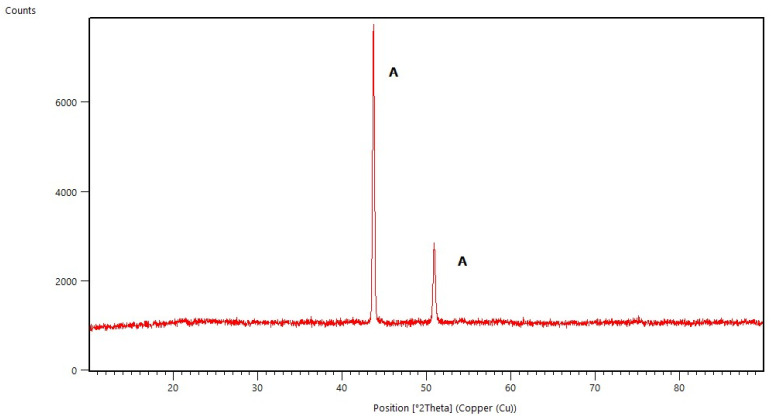
X-ray Diffraction pattern of basic TEOS thin film deposited on steel (A—austenite).

**Figure 2:**
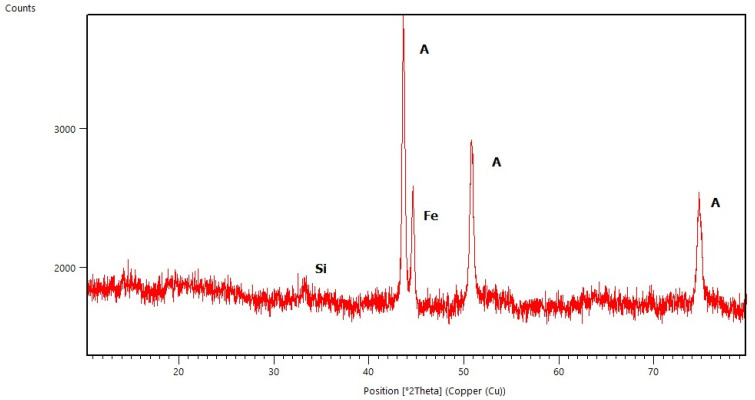
X-ray Diffraction pattern of Aerosil^TM^ thin film deposited on steel (A—austenite, Fe—iron, Si—silica (undefined)).

**Figure 3:**
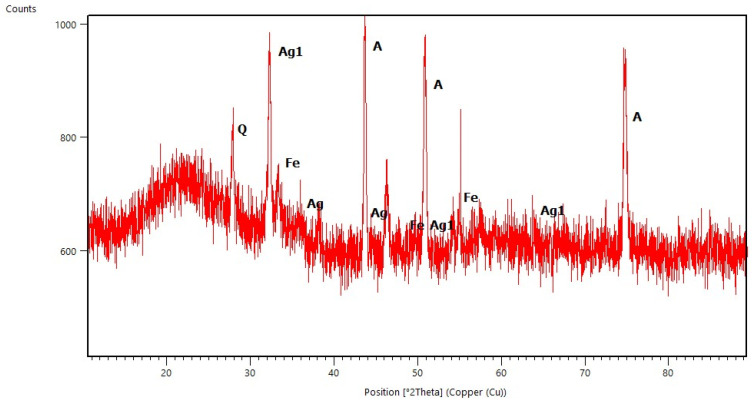
X-ray Diffraction pattern of basic TEOS/Ag thin film deposited on steel (A—austenite, Ag1—AgO, Ag—Ag, and Fe—iron).

**Figure 4:**
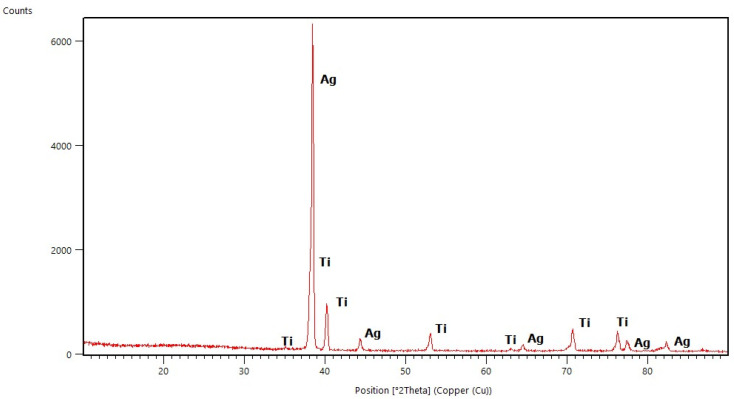
X-ray Diffraction pattern of basic TEOS/Ag thin film deposited on titanium (Ti—titanium and Ag—silver).

All diffraction patterns were collected for the coatings annealed at 500 °C for 30 min. The measurements of coatings without Ag addition allowed us to observe the amorphous state of the deposited layers (selected diffraction patterns are presented in Figure 1, Figure 2, Figure 3 and Figure 4) and to distinguish the reflections originating from the substrates. These are the main peaks caused by austenite (00-023-0298 PDF2 identification card) at approximately 43 °C and 51 °C [2θ] for steel substrates, and at 35 °C, 40 °C, 53 °C, 76 °Cand 78 °C [2θ] for titanium (01-089-2762 PDF2 identification card). In some diffraction patterns, e.g., for Aerosil^TM^ deposited on steel (Figure 3), one can distinguish very weak peaks due to the silica phase but not any defined polymorph type.

When silver atoms were introduced, in the diffraction patterns of thin films (Figure 3 and Figure 4) one could observe peaks characteristic of the silver phase (01-076-149, 01-089-3722 PDF2 identification cards). Similarly to the measurements of samples without Ag addition, it was also possible to distinguish the peaks assigned to the silica phase structure, and—in the case of the TEOS/Ag layer deposited on steel (Figure 3)—additionally the ones connected to the first traces of the quartz structure. This can point to silver playing the role of a crystallization nucleus as found in other systems with Ag addition [11].

The presence of peaks due to the silver phase allowed us to calculate the average sizes of Ag crystallites based on FWHM (Full Width at Half Maximum) of selected peaks and the Scherrer Equation (4). The results are collected in Table 1. All crystallites were smaller than 100 nm so they can be considered to be nanoparticles of Ag. Moreover, the average sizes of Ag crystallites observed in coatings deposited on titanium were bigger than those identified in coatings deposited on steel. There may be two reasons for this phenomenon. First annealing at 500 °C leads to increased crystallization of the studied samples. As the second reason, the rate of polycondensation and polymerization processes of sols/gels can be proposed.

### 2.2. IR Spectroscopy Studies

**Figure 5:**
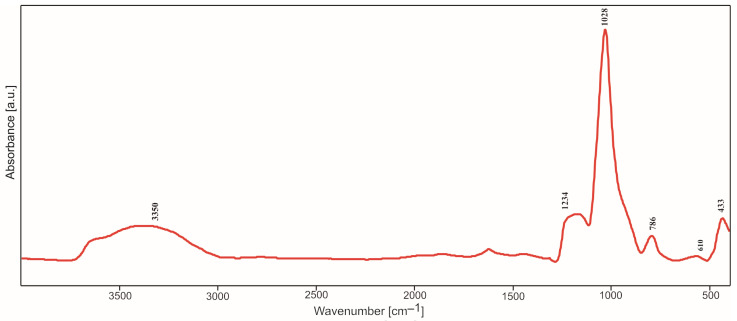
IR spectrum of basic TEOS thin film deposited on steel.

**Figure 6:**
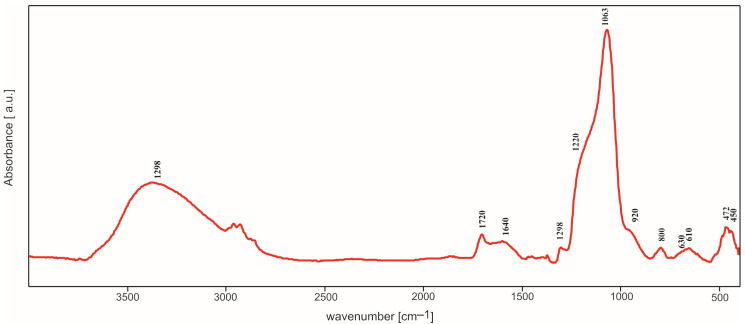
IR spectrum of TEOS/DDS thin film deposited on steel.

**Figure 7:**
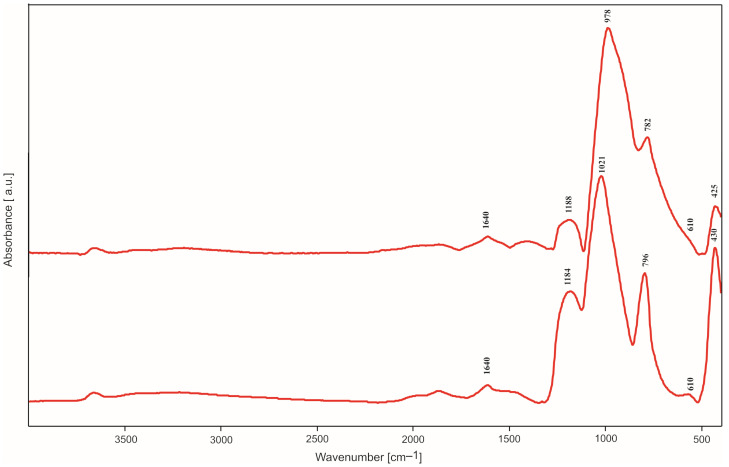
IR spectra of basic TEOS/Ag thin films, deposited on titanium (top) and steel (down) substrates.

**Figure 8:**
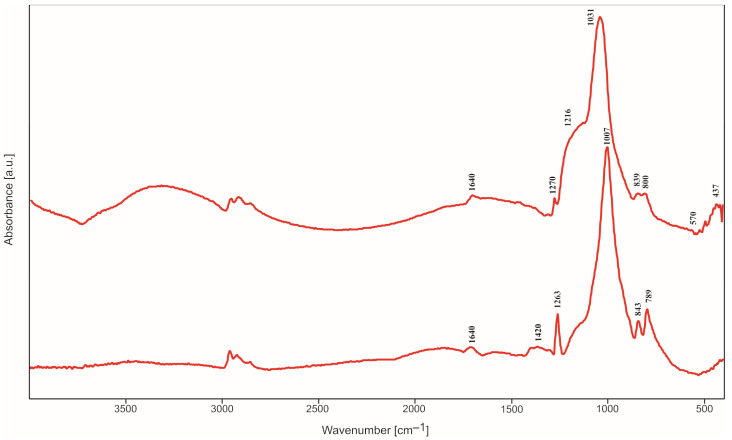
IR spectra of TEOS/DDS/Ag thin films deposited on steel (top) and titanium (down) substrates.

All IR spectra (selected spectra are presented in Figure 5, Figure 6, Figure 7 and Figure 8) were measured in the reflectance mode, for the samples initially annealed at 500 °C in air (steel substrates) or argon (titanium substrates). Despite heating at 500 °C, the band characteristics of OH group stretching and bending vibrations in water molecules appear in the range of 3000–3700 cm^−1^ and at 1650 cm^−1^, respectively, in all the obtained IR spectra. As expected for the samples containing silica, four main bands assigned to Si-O bond vibrations can also be easily distinguished, at approximately 430 cm^−1^, 790 cm^−1^, 1010 cm^−1^ and 1210 cm^−1^. These bands are observed in all IR spectra, independently of the type of silica precursor and substrate. However, their intensities are influenced by the SiO_2_ precursor and substrate. The most intensive band in all the spectra, at approximately 1010–1038 cm^−1^, can be assigned to the asymmetric stretching vibrations of Si-O-Si bridges while the band at 790–800 cm^−1^ can be assigned to the symmetric ones [12]. The band and a shoulder at 1210–1220 cm^−1^ are probably due to the double Si=O bond vibrations.

The presence of this band can be related to the type of synthesis and content of the amorphous phase in the samples, which is confirmed by the XRD studies. The weak bands in the range of 430–470 cm^−1^ are ascribed to O-Si-O bending vibrations. There is an additional band (or shoulder), which appears between 830 and 920 cm^−1^. It can be assigned to the vibrations of Si-O^−^ broken bridges and its intensity depends on the type of silica precursor and the substrate.

In the spectra of thin films synthesized with DDS, there is a band at 1268–1290 cm^−1^ which can be due to the vibrations of C-H bonds in Si-CH_3_ groups incorporated into the structure of thin films. The weak band at approximately 570–610 cm^−1^ observed in the spectra of thin films containing silver can be caused by the incorporation of non-tetrahedral cations (e.g., Ag^+^) into the studied structure. This band can be treated as a kind of Ag atoms/ions presence marker [11] but it is ususally not observed when Ag concentration is extremely low.

### 2.3. Raman Spectroscopy Studies

**Figure 9:**
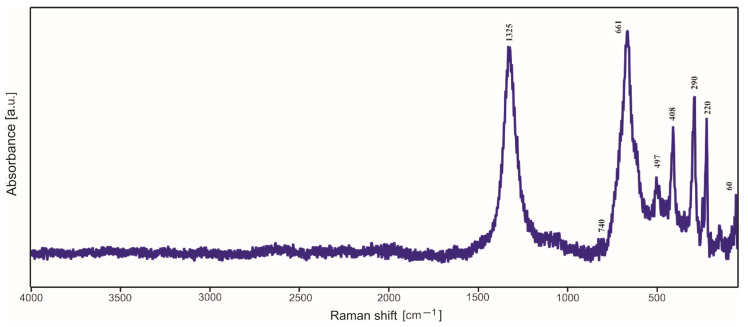
Raman spectrum of basic TEOS thin film deposited on steel.

**Figure 10:**
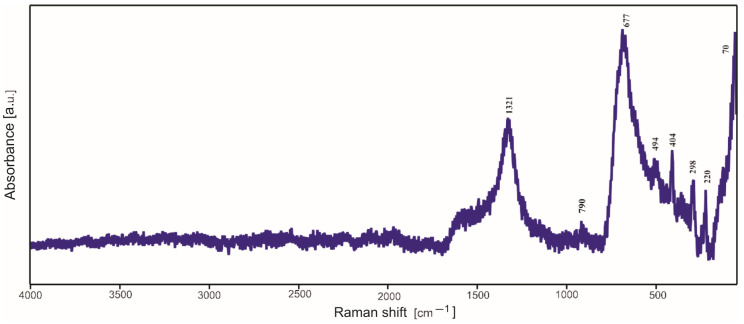
Raman spectrum of TEOS/DDS/Ag thin film deposited on steel.

**Figure 11:**
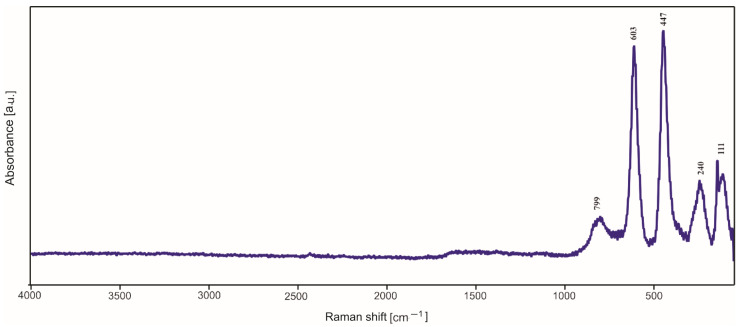
Raman spectrum of Aerosil^TM^ thin film deposited on titanium.

In most cases, in the Raman spectra (Figure 9, Figure 10 and Figure 11), it is not possible to distinguish the bands due to the amorphous silica, presence of which was established by XRD investigations. Moreover, the strong signal originating from the substrates of the samples overlaps with the weak signal of a silica structure. However, after the detailed analysis, one can distinguish the weak band with a maximum at 740–799 cm^−1^, which can be assigned to the bending vibrations of the Si-O bond. Other bands are due to the products of surface corrosion, which probably started during the annealing of deposited thin films in air but quite unexpectedly also in argon (for titanium substrate).

The bands at 220 cm^−1^, 290–296 cm^−1^, 408 cm^−1^, 494–497 cm^−1^ and 1325 cm^−1^ observed in the Raman spectra of thin films deposited on steel (Figure 9 and Figure 10) are characteristic of hematite Fe_2_O_3_, while the strongest band at 661–670 cm^−1^ can be assigned to magnetite. Both hematite and magnetite are corrossion products which can occur on the surface of steel.

### 2.4. SEM Studies

**Figure 12:**
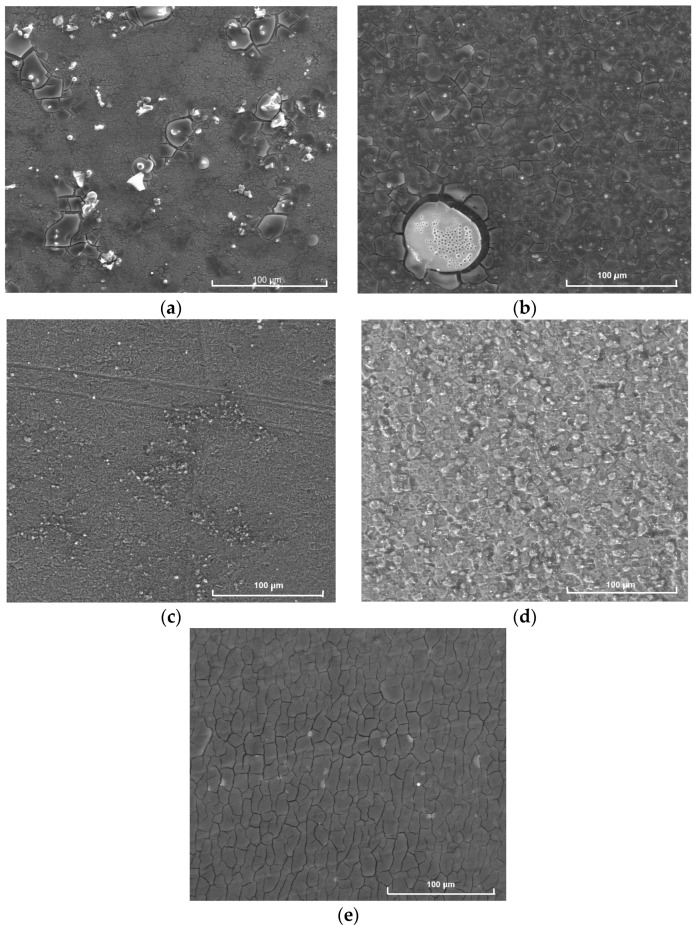
SEM images of: (**a**) of basic TEOS thin film deposited on steel; (**b**) basic TEOS/Ag thin film deposited on titanium; (**c**) TEOS/DDS thin film deposited on steel; (**d**) TEOS/DDS/Ag thin film deposited on titanium; (**e**) Aerosil^TM^/Ag thin film deposited on titanium.

SEM imaging of thin films surface was performed for all deposited coatings. The surface images of selected but characteristic samples are presented in Figure 12. EDS results of the examined samples are collected in Table 2. All thin films obtained by dip coating technique contain a network of cracks located all over their surface, probably caused by heat treatment of the samples at 500 °C, in air as well as in argon. The cracks are not deep and thus the coatings are tight and adhere well to the substrate. It is also important that each coating consists of six separately deposited layers, which enhances their tightness. The main difference between the coatings synthesized with TEOS and with TEOS and Ag addition is the presence of oval porous objects in layers containing silver (Figure 12a,b). These objects originate probably from the decomposition of AgNO_3_ under the influence of temperature and were observed also by other scientists [13].

Thin films synthesized with TEOS and DDS show more compact and dense structure (Figure 12c,d), which indicates a strong influence of this silane on the surface topography, more significant than that caused by Ag addition. Moreover, although Ag was introduced using AgNO_3_, large oval objects, as previously mentioned, are not observed in thin films prepared with both Ag and DDS (Figure 12d). The most regular network of cracks is observed in the case of the thin film deposited using the sol synthesized with Aerosil^TM^ and AgNO_3_ (Figure 12e). The topography of thin films obtained using fume silica was similar independently of the type of substrate.

EDS results confirm the presence of Ag in coatings synthesized using AgNO_3_ on both steel and titanium substrates (Table 2). The differences in the Ti concentration in the thin films deposited on the same substrate are strongly associated with different thicknesses of the coatings. The reason for differences in the composition of coatings on steel substrates (Table 2) is exactly the same, i.e., their different thicknesses in the areas/points of studies.

### 2.5. AFM Studies

**Figure 13:**
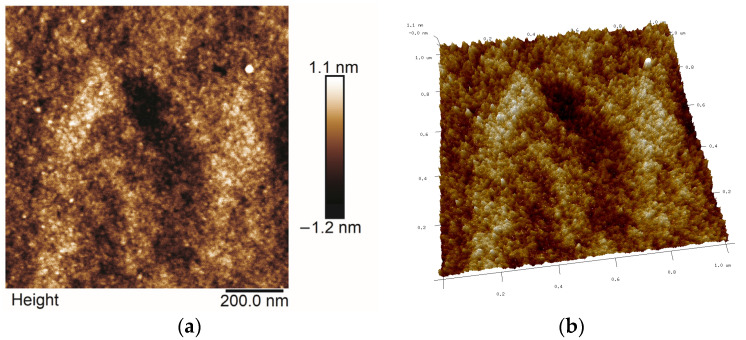
2D (**a**) and 3D (**b**) AFM microphotographs of basic TEOS thin film deposited on steel.

**Figure 14:**
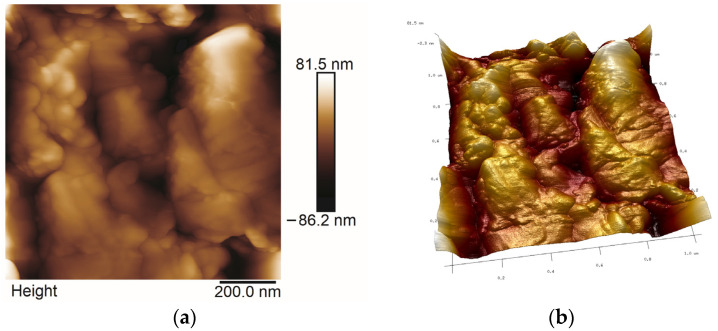
2D (**a**) and 3D (**b**) AFM microphotographs of TEOS/DDS/Ag thin film deposited on steel.

**Figure 15:**
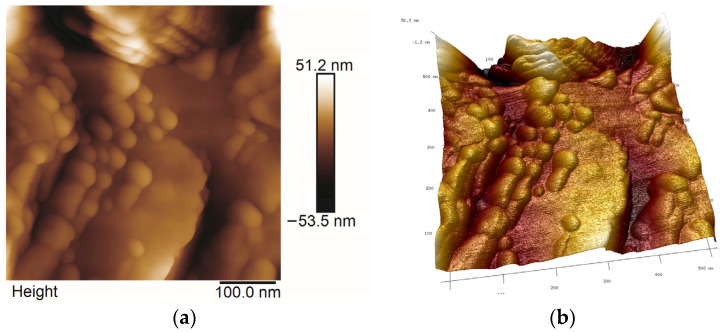
2D (**a**) and 3D (**b**) AFM microphotographs of TEOS/DDS/Ag thin film deposited on titanium.

**Figure 16:**
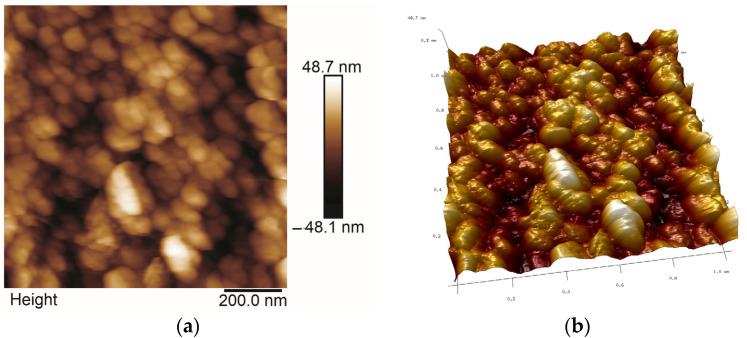
2D (**a**) and 3D (**b**) AFM microphotographs of Aerosil^TM^/Ag thin film deposited on steel.

All AFM images were obtained in the Tapping or Peak Force Tapping mode. The selected ones are presented in Figure 13, Figure 14, Figure 15 and Figure 16. Roughness parameters R_a_, R_q_ and R_max_ were calculated using scans of 1 μm × 1 μm size (Table 3).

Analyzing the topography of surface of coatings on the nanoscale, one can notice that the sizes of Ag particles and the roughness of thin films are different, which was not seen so distinctly in SEM images. The smallest R_a_ values were obtained for the thin film synthesized with TEOS (Figure 13) while the highest R_a_ was calculated for thin films deposited on steel from Aerosil^TM^ and Ag solution (Figure 16), (Table 3). The surface of thin films synthesized with TEOS, DDS and containing Ag contained large agglomerates and much smaller spherical particles of average dimensions of 20–40 nm (Figure 14 and Figure 15). These values agree with Ag crystallites sizes calculated from the Scherrer formula for the same samples (Table 1). Therefore, it is likely that these spherical objects are silver particles.

Coatings synthesized with Aerosil^TM^ alone or with Ag exhibit the highest roughness because they contained large formations consisting of few particles (Figure 16), creating very porous structures, which can further influence the hydrophilicity of these samples. The roughness of the sample surface, especially nano roughness, given by the R_a_ and R_q_ parameters, can also influence the adhesion of the deposited coatings to the substrate, local corrosion processes and hydrophilicity/hydrophobicity of the surface of samples.

### 2.6. Wetting Angle Value Measurements

**Figure 17:**
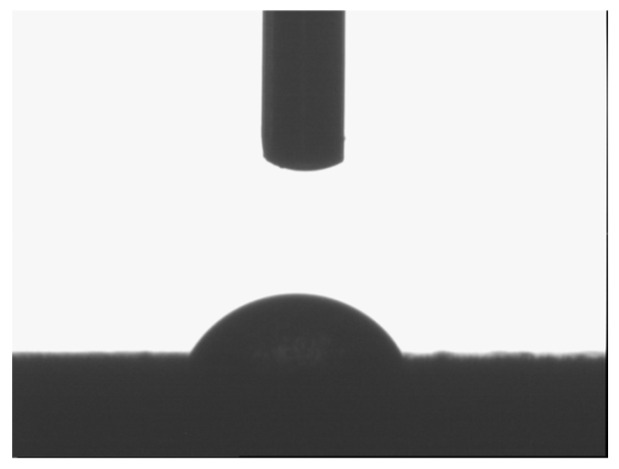
The example of the sessile drop method measurement.

The measurements of wetting angle values enable estimating the hydrophilic or hydrophobic properties of samples. To run such measurements, the method of sessile drop was applied (Figure 17).

The deionized water was used to measure wetting angle values. The measurements were performed 10 times for each sample. The results are presented in Table 4.

The hydrophilicity of thin films synthesized with Aerosil^TM^ was so high that wetting angle values were not measurable because the surfaces of samples behaved like a sponge. Coatings obtained with TEOS, DDS and with Ag addition exhibited wetting angle values over 90 °C, which points to hydrophobic nature of these samples. Thin films deposited on steel showed almost the same values of the wetting angle at 53–58 °C, which proves the hydrophilic properties of these samples. Pure titanium and steel substrates show the wetting angle values between 75 and 90 °C, which also indicates their hydrophilic properties.

## 3. Discussion

The main goal of this work was to determine the influence of different types of silica precursors and Ag addition on the nano/microstructure and topography of deposited thin films. The second examined problem was to verify if the applied substrate influenced the structure of coatings.

According to the XRD (in GID configuration) studies, all deposited thin films remained amorphous even after annealing at 500 °C, which strongly influenced the distribution of properties through the whole structure of the samples. The crystalline reflections observed in the diffraction patterns were mainly due to phases present in the substrates. The only crystalline phase observed in the samples and originating from thin films was silver. The sizes of Ag crystallites were in the range of nanoparticles (Table 1) and they were dispersed in the silica matrix as was also described in [14].

These observations were confirmed by IR studies. In the IR spectra, one could observe first of all the bands associated with the presence of Si-O bonds (Figure 5, Figure 6, Figure 7, Figure 8, Figure 9, Figure 10 and Figure 11). These were two bands due to the asymmetric and symmetric stretching vibrations of Si-O bonds, at approximately 1020 cm^−1^ and 790–800 cm^−1^, respectively, as well as one band assigned to the bending vibrations of O-Si-O linkages at 430–470 cm^−1^ [12,14]. There was also one additional band at approximately 1180–1200 cm^−1^ ascribed to vibrations in Si=O bonds. The intensity of this band increased as Ag addition grew, which points to the depolymerization of silica structure (Figure 7), but simultaneously it decreased with DDS addition (Figure 6 and Figure 8), which could be connected to the opposite effect. In the IR spectra of thin films synthesized with DDS, one could distinguish another two bands, one at 1260–1290 cm^−1^ due to the vibrations of C-H bonds in CH_3_ groups [15,16] and one at 830–920 cm^−1^ assigned to Si-O^−^ broken bridges. The band at approximately 1260 cm^−1^ confirmed the occurrence of methyl groups despite the annealing of coatings at 500 °C. The presence of the band at 920 cm^−1^ pointed to the predominance of the depolymerization process of silica lattice over polymerization, which was more pronounced in Ag-containing thin films. The weak band at approximately 570–610 cm^−1^ (observed in the IR spectra of the samples containing silver) could be connected to the presence of the non-tetrahedral Ag^+^ ions. This band is usually observed in the samples of higher Ag concentrations [11].

The homogeneity of thin films surfaces was confirmed by SEM observations. SEM images showed a similar network of cracks in all samples (Figure 12), with the only exception of coatings synthesized with TEOS and containing Ag, where oval, porous objects were visible, probably as the result of AgNO_3_ thermal decomposition. Despite these observations, coatings showed good adhesion and tightly covered the substrates. More detailed information on the topography of the samples was provided by AFM microscopy (Figure 13, Figure 14, Figure 15 and Figure 16). AFM nanoscaled images of coatings showed differences in the sizes of particles and in the roughness of the samples. Generally, thin films deposited on steel showed higher roughness than those obtained on titanium substrate. The influence of Ag on roughness parameters is not so distinct as the influence of substrate type. The thin films obtained with Aerosil^TM^ showed the highest roughness of all studied samples and behaved like sponge during the wetting angle measurements, making it impossible to carry out these measurements. The wetting angle values of all other samples pointed to their hydrophilic properties, like pure steel and titanium. The only exception was the thin film synthesized with TEOS, DDS and containing Ag, which has a wetting angle value of 106.18 °C, indicating its hydrophobic character, which could be connected to the influence of methyl groups originating from DDS structure [15].

All the synthesized thin films showed good adhesion to the substrate and, in spite of the presence of the network of cracks, tightly covered the substrates because each coating consisted of six layers deposited in three processes and annealed after each two depositions. As amorphous materials, with Ag nanoparticles dispersed in the silica matrix (in the case of samples containing silver), they should be homogeneous and exhibit uniform distribution of properties in their structure. Comparing all three silica precursors, the strongest influence on the structure and properties of the samples was observed in the case of Aerosil^TM^. Ag addition and the type of substrate did not affect the structure and properties of the samples as significantly as Aerosil^TM^.

## 4. Materials and Methods

Three different SiO_2_-containing sols were prepared using three different silica precursors. As the precursors, TEOS—tetraethoxysilane Si(OC_2_H_5_)_4_ (Aldrich 98%), DDS—dimethyldiethoxysilane (CH_3_)_2_(C_2_H_5_O)_2_Si (Aldrich 97%) and Aerosil^TM^ (fumed silica, Sigma-Aldrich) were selected. TEOS belongs to the group of commonly used silanes, which, together with their pH value and the catalyst applied, determine the rate of hydrolysis and polycondensation of sols prepared with it [17,18,19]. The use of DDS in the synthesis can increase gelation time as well as hydrolysis and polycondensation rates [13,15,20,21,22]. Because of the presence of methyl groups in its structure, DDS can influence the hydrophobicity of samples as well as their antireflective properties. Silver atoms were introduced into the sols using silver nitrate AgNO_3_ (Chempur 99.9%) as their source.

The sol–gel process, in which Si alkoxides—M(OR)_n_ (where M can mean a metal, Si in this case, whereas R means alkyl group in an alkoxy group -OR) are applied, is described by three main reactions (1), (2) and (3).

The hydrolysis of the M-OR alkoxy group results in the formation of a M-OH bond according to the following reaction:

M(OR)_n_ + H_2_O ↔ (RO)_n−1_M − OH + ROH (1)

When a sol is transformed into a gel, the condensation between hydroxy and alkoxy (2) or two hydroxy (3) groups takes place:

(RO)_n−1_M − OH + M(RO)_n_ ↔ (RO)_n−1_M − O − M(RO)n − 1 + ROH (2)

(RO)_n−1_M − OH + HO − M(RO)_n−1_ ↔ (RO)_n−1_M − O − M(RO)_n−1_ + HOH (3)

In reactions (2) and (3), M − O − M bridges are formed, which creates a kind of skeleton of the structure of the synthesized material. In our case, it is the Si-O-Si structure of the synthesized samples.

**Figure 18:**
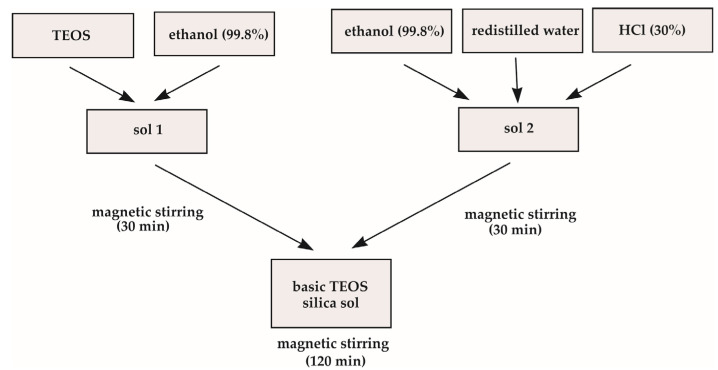
The scheme of first (basic) silica sol synthesis.

To obtain the first silica sol, two raw solutions were prepared, one containing TEOS and ethanol (99.8%) as solvent and the second one, containing ethanol (99.8%), redistilled water and HCl (30% Fluka) as catalyst (Figure 18). Appropriate amounts of all components were taken to obtain the assumed molar ratios, TEOS:H_2_O = 1:4 and TEOS:HCl = 4:1. Both separate solutions were then stirred for 30 min each. After that, the second solution was added dropwise to that containing TEOS, stirred for 2 h, and then refrigerated.

**Figure 19:**
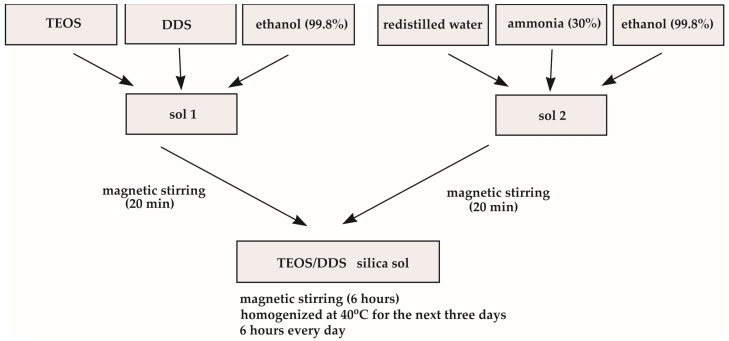
The scheme of second TEOS/DDS sol synthesis.

The second sol was synthesized using TEOS and DDS at TEOS:DDS = 1:1 molar ratio (Figure 19). Other components were introduced in the amounts that ensured DDS:TEOS:H_2_O:C_2_H_5_OH:NH_4_OH = 1:1:3:80:0.1 molar ratio [15,20]. At the first stage, the sol containing ethanol (99.8%), TEOS and DDS was prepared and homogenized for 20 min. Simultaneously, a solution of ethanol (99.8%), redistilled water and ammonia (30% Fluka—Honeywell, Charlotte, NC, USA) was stirred for the same time. After that, a solution of TEOS and DDS in ethanol was slowly added to the solution of ammonia in ethanol and water, the obtained sol was then stirred for 6 h. After that, it was homogenized at 40 °C during the next three days, 6 h each day. The obtained sol was then divided into two parts, one of them was homogenized (sonicated) in Sonics VibraCell (Sonics and Materials, Newtown, CT, USA) and both were then refrigerated until being used for deposition onto a substrate.

**Figure 20:**
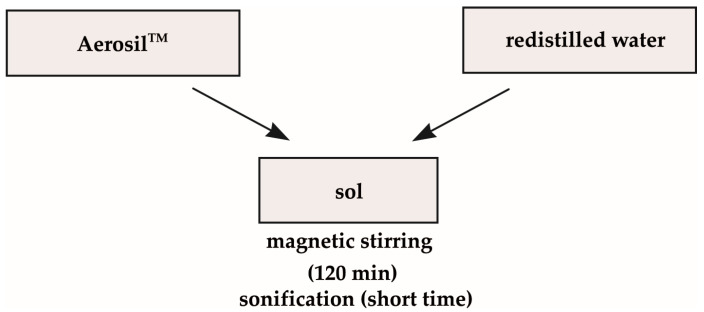
The scheme of synthesis of third sol using Aerosil^TM^ as silica precursor.

The third sol was obtained by dissolution of 9.77 g of Aerosil in 270 mL of redistilled water and later stirred for 2 h (Figure 20). After that, the prepared sol was sonicated for a short time.

The second series of the sols was prepared in order to incorporate Ag atoms into the coatings. AgNO_3_ served as a silver source. The appropriate amounts of AgNO_3_ were added to each sol during the synthesis to obtain Ag:Si ratios collected in Table 1. All containers with sols were then kept with no access to light because of the presence of the photosensitive component.

Thin films were deposited on two types of substrates: steel and titanium (Table 5). Before deposition of coatings, the substrates in the form of rectangles of dimensions 10 × 20 mm were thoroughly cleansed with sandpaper and degreased with acetone, then rinsed with redistilled water and finally dried. Coatings were deposited using the home made equipment by dip-coating technique, at the speed of 4 cm/min of immersion and emergence. Such a procedure was repeated six times but after every two depositions, the samples were heated at 500 °C in air (steel substrate) or argon (titanium one) for 30 min.

As mentioned in Section 4, the main goal of this work was to determine the influence of different types of silica precursors and Ag addition on the topography and a nano/microstructure of deposited thin films. To achieve this goal, two methods of structure investigations were selected: IR spectroscopy and X-ray Diffraction, also in GID (Grazing Incidence Diffraction) configuration. The data obtained by both methods provided complete information on the far and near order in the samples of ordered or disordered lattice.

IR spectra were collected on a Bruker 70 V IR spectrometer (Bruker, Billerica, MA, USA) at a resolution of 4 cm^−1^ while all diffraction patterns were collected on an X’Pert Diffractometer (Panalytical, Almelo, The Netherlands) using CuK_α_ radiation. All data graphs were obtained with the aid of the software supplied by equipment manufacturers. The average sizes of silver particles were calculated using the Scherrer equation:

(4) $$D_{hkl}=\frac{k\lambda }{\beta cos\theta }$$

where

*β—*the full width at half maximum, *β = β_obs_ − β_stand_*, [rad];
*λ—*the wavelength of radiation applied (CuK_α_), *λ* = 0.15406 [nm];
*θ—*the peak position [°];
*k—*Scherrer constant, *k* = 0.9;
*D_hkl_*—the average crystallite size perpendicular to the plane, which gave the reflection [nm].

As the additional structure research methods, Raman spectroscopy and for the surface imaging, SEM (Scanning Electron Microscopy) and AFM (Atomic Force Microscopy) microscopy were selected. Raman spectra were measured on a HORIBA LabRAM HR spectrometer (HORIBA France SAS, Longjumeau, France) using a Nd:YAG laser (532 nm). SEM studies were performed on a NOVA NANO SEM 200 microscope (FEI EUROPE B.V.Company, Eindhofen, The Netherlands) while AFM was performed on a Multimode 8 atomic force microscope (Bruker, Billerica, MA, USA) using the Force Tapping mode.

The hydrophobicity or hydrophilicity of the samples was estimated by wetting angle value measurements carried out on a DSA 10 Kruss goniometer (A. Krüss Optronic GmbH, Hamburg, Germany), by the sessile drop method.

## Figures and Tables

**Table 1:** Sizes of Ag crystallites.

Sample	*D_hkl_* (Average Crystallites Size) [nm]
basic TEOS/Ag on steel	20.5
basic TEOS/Ag on titanium	24.0
TEOS/DDS/Ag on steel	15.2
TEOS/DDS/Ag on titanium	20.5
Aerosil^TM^/Ag on titanium	23.5

**Table 2:** EDS results of the selected studied samples.

Sample	Elements													
	Thin Film						Substrate							
	O		Si		Ag		Ti		Cr		Fe		Ni	
	Wt%	At%	Wt%	At%	Wt%	At%	Wt%	At%	Wt%	At%	Wt%	At%	Wt%	At%
TEOS/steel	24.72	47.72	18.82	20.70	-	-	-	-	11.18	6.64	41.29	22.84	4.00	2.10
TEOS/Ag/Ti	45.03	66.66	21.75	18.34	5.20	1.14	28.02	13.86	-	-	-	-	-	-
TEOS/DDS/ steel	10.63	28.39	3.55	5.40	-	-	-	-	14.96	12.30	62.83	48.07	8.02	5.84
TEOS/DDS/Ag/Ti	24.86	49.22	4.72	5.33	3.03	0.89	67.39	44.56	-	-	-	-	-	-
Aerosil^TM^/Ag/Ti	49.27	69.35	24.25	19.44	4.77	1.00	21.71	10.21	-	-	-	-	-	-

**Table 3:** Roughness R_a_, R_q_ and R_max_ parameters of samples.

Sample	R_a_ [nm]	R_q_ [nm]	R_max_ [nm]
basic TEOS on steel	0.335	0.263	4.65
TEOS/DDS on steel	20.9	27.9	210
TEOS/DDS/Ag on titanium	9.93	13.4	127
TEOS/DDS/Ag on steel	17.3	0.224	169
Aerosil^TM^ on titanium	10.5	13.2	92.2
Aerosil^TM^/Ag on steel	44.1	55.8	345

**Table 4:** Wetting angle values of synthesized thin films and substrates.

Sample	Substrate	Wetting Angle [°]	Standard Deviation [°]
Basic TEOS	steel	53.53	6.96
Basic TEOS/Ag	steel	58.73	8.94
Basic TEOS/Ag	titanium	86.38	7.35
TEOS/DDS/Ag	steel	41.75	14.31
TEOS/DDS/Ag	titanium	106.18	4.53
Aerosil^TM^	steel	Not measurable	
Aerosil^TM^	titanium	Not measurable	
-	steel	89.94	7.19
-	titanium	75.74	12.03

**Table 5:** Prepared samples—types of sols, substrates and Ag addition.

Sample/sol	Steel	Titanium	Ag Addition Ag:Si Ratio [mol]
basic	x	-	-
basic	-	x	-
basic	x	-	1:12
basic	-	x	1:12
TEOS/DDS	x	-	-
TEOS/DDS	-	x	-
TEOS/DDS	x	-	1:70
TEOS/DDS	-	x	1:70
Aerosil	x	-	-
Aerosil	-	x	-
Aerosil	x	-	1:85
Aerosil	-	x	1:85

## Data Availability

The raw data supporting the conclusions of this article will be made available by the authors on request.
